# Mycotic aortic aneurysms post-Intravesical BCG treatment for early-stage bladder carcinoma

**DOI:** 10.1186/s42155-018-0036-y

**Published:** 2018-08-22

**Authors:** Aman Wadhwani, Randy D. Moore, Darshan Bakshi, Anirudh Mirakhur

**Affiliations:** 1 Department of Radiology, University of Calgary, Calgary, AB Canada; 2 Division of Vascular Surgery, Department of Surgery, University of Calgary, Calgary, AB Canada; 3 Division of Interventional Radiology, Department of Radiology, University of Calgary, Calgary, AB Canada; 4 Foothills Medical Centre, G29, 1403-29th street NW, Calgary, AB T2N 2T9 Canada

**Keywords:** Mycotic aortic aneurysms, BCG

## Abstract

**Background:**

Intravesicular Bacillus Calmette-Guérin (BCG) is an effective adjunctive therapy for superficial bladder cancer that has been shown to delay recurrence and progression of disease. Serious side effects are relatively rare but are difficult to diagnose and are often overlooked. Vascular complications are particularly rare.

**Case presentation:**

We report two cases of mycotic aortic aneurysms secondary to BCG treatment, one managed with endovascular stent-graft placement and the other with open surgical repair. The present understanding of disseminated BCGosis, including a literature review, will be discussed.

**Conclusion:**

The incidence of mycotic aneurysms from BCG treatment is rare and very few cases have been reported in the literature. These cases further expand the current knowledge on vascular complications related to BCG treatment. In the absence of formal guidelines, we recommend a multidisciplinary approach involving vascular surgery, diagnostic and interventional radiology, and infectious disease to manage these patients.

## Background

Bacillus Calmette-Guérin (BCG) is a live attenuated strain of *Mycobacterium bovis* (M bovis) that was initially developed for vaccination against tuberculosis. Several randomized controlled trials have demonstrated that intravesical instillation of BCG reduces the recurrence of high risk superficial transitional cell carcinoma (TCC) (Shelley et al., 2004). Although side effects of BCG therapy have been described in the literature, serious complications are rare. The most common side effects are symptoms of cystitis such as dysuria and urinary frequency that develop in approximately 70% of patients (Lamm et al., 1992). A sepsis-like syndrome with fever, hypotension, and respiratory failure occurring in 0.04% of patients likely represents a hypersensitivity reaction to BCG as opposed to an infection. Although an infection due to BCG dissemination is rare, granulomatous hepatitis/pneumonitis (0.7%) and prostatitis (0.9%) have been reported (Lamm et al., 1992).

M bovis associated aortitis causing a mycotic aortic aneurysm (MAA) is extremely rare and has been reported in less than 30 cases to date. In this article, we report 2 cases of mycotic aortic aneurysms in patients that received recurrent BCG instillations as a part of routine therapeutic regimen for their early T1N0M0 TCC.

## Case presentation

### Patient A

**Fig. 1:**
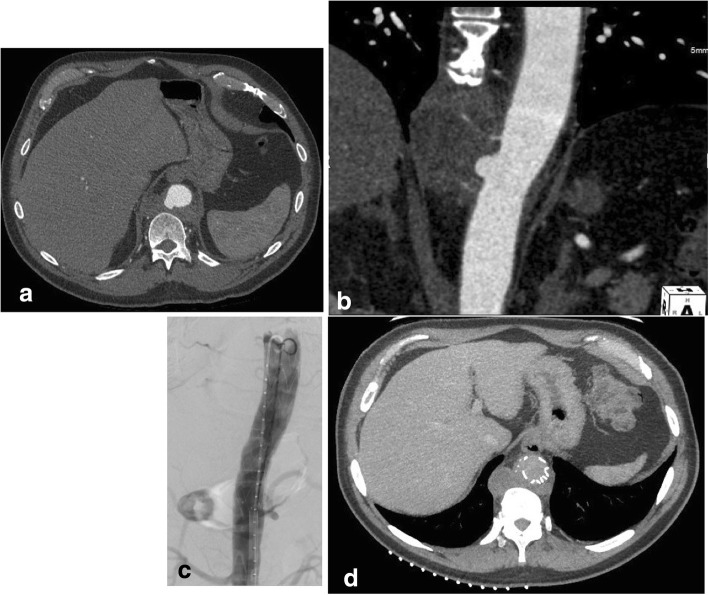
Sixty seven year old male with mycotic aneurysm of the thoracic aorta. **a** Axial and **b** coronal images show enhancing, multiseptated, and lobulated low density mass within the lower retrocrural space/posterior mediastinum that abuts the adjacent thoracic aorta along 180 degrees of contact. Focal outpouching of the descending thoracic aorta at this site is consistent with a psuedoaneurysm. **c** Post stent graft deployment angiogram and **d** CT show satisfactory placement of the stent graft with no residual psuedoaneurysm or post procedural endoleaks

Patient A was a 73-year-old male who presented to the emergency department (ED) with abdominal pain and low-grade fevers. He presented 6 months after the last of his five intravesical BCG instillations for his known non-muscle invasive urinary bladder papillary TCC. His medical profile included COPD, Type II diabetes, hypertension, dyslipidemia, and macular degeneration. There was history of remote TB exposure in childhood with no treatment or related hospital admissions. In ED, his complete blood cell count was within normal limits. Serum C-reactive protein was elevated at 58.6 mg/L (normal: 0–8.0 mg/L). Initial cross sectional imaging at the time of presentation demonstrated a new, multi-septated peripherally enhancing 6.3 cm × 1.9 cm × 5.6 cm, low-density collection within the retrocrural/posterior mediastinal region abutting the descending thoracic aorta along 180 degrees of circumference of the vessel (Fig. 1). Along the right posterolateral wall, an enhancing focal outpouching arising from the descending thoracic aorta was also identified. On positron emission tomography/computed tomography (PET/CT), this lesion demonstrated peripheral intense hypermetabolism with central photopenia. PET/CT did not demonstrate any additional hypermetabolic lesions and was negative for tumor recurrence or metastatic disease elsewhere. A follow-up MRI of the thoracic spine was negative for discitis or osteomyelitis. CT-guided aspiration of the retrocrural abscess yielded mycobacterium bovis consistent with BCG on pathology. Given the constellation of findings and pathology results, the patient was treated with Isoniazid, rifampin, pyrazinamide, ethambutol, and Vitamin B6. Given the patient’s medical comorbidities, the mycotic aneurysm identified on CT was treated with endovascular stent graft placement as opposed to open surgical repair. No post-procedural complications were identified on CT. Adjunctive percutaneous drainage of the periaortic collection was also performed. Subsequently, over a course of 6 months the patient has remained asymptomatic, although the lesion has shown no signs of regression.

### Patient B

**Fig. 2:**
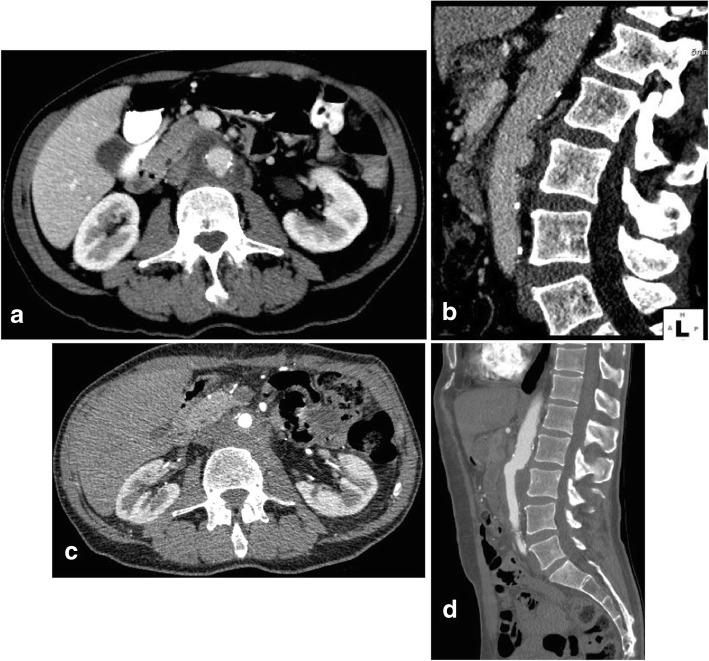
Seventy three year old male with mycotic aneurysm of the abdominal aorta. **a** Axial and **b** sagittal images of a portal venous phase exam showing peripherally enhancing hypoattenuating periaortic mass with a focal out pouching arising from the posterior wall of the aorta consistent with a psuedoaneurysm. Post-surgical repair **c** axial and **d** sagittal images of CTA show unremarkable superficial femoral vein graft with no residual psuedoaneurysm

Patient B was a 67-year-old male who presented to ED with weight loss and night sweats. His complete blood cell count was within normal limits. Serum C-reactive protein was elevated at 41.1 mg/L. He had received fifteen intravesical BCG instillations for his low-grade papillary urothelial carcinoma and his last BCG instillation was 4 months prior to his ED presentation. His medical profile was otherwise negative apart from a remote uncomplicated appendectomy. Outpatient abdominal ultrasound was suspicious for periaortic lymphadenopathy. Follow-up CT scan of the abdomen and pelvis showed a centrally low density and peripherally enhancing periaortic collection measuring up to 5 cm × 1 cm × 4.6 cm in the infrarenal region. At this level, a focal outpouching arising from the aorta posteriorly measuring up to 0.8 cm × 2.2 cm × 0.9 cm (APxTRVxCC) was suspicious for a mycotic aneurysm (Fig. 2). A percutaneous CT-guided biopsy of the periaortic collection demonstrated necrotizing granulomatous inflammation, highly suspicious for BCGosis. This patient’s mycotic aneurysm was treated with a surgical resection of the infected infrarenal aortic segment and repaired using an autologous graft harvested from the patient’s left femoral vein. He had an uneventful postoperative course in the hospital and was discharged on a standard antituberculosis medication regime including isoniazid, rifampin, pyrazinamide, ethambutol, and Vitamin B6. Patient has remained asymptomatic awaiting six-month follow-up imaging studies.

## Discussion

Intravesical BCG injection following transurethral resection (TUR) is the standard of care for treating high-risk superficial bladder cancer (Shelley et al., 2000). BCG decreases the likelihood of tumor recurrence and progression after TUR (Shelley et al., 2000; Lamm, 2000). The antitumor effect of BCG is driven by its T-cell mediated inflammatory response. This inflammatory response is also hypothesized to cause iatrogenic cystitis with dysuria, urinary frequency and low grade fever.

MAA following BCG treatment are rare. The overall incidence of MAA is also relatively low and account for 0.7% to 4.5% of all aortic aneurysms (Oderich et al., 2001). Both our patients presented with vague constitutional symptoms such as epigastric discomfort, weight loss, low grade fevers, and night sweats. Importantly, their presentation to ED was, 6 months and 4 months respectively, after their last BCG instillations. As discussed, CT exams in both cases at the time of emergency presentation showed irregular centrally cystic and peripherally enhancing periaortic collections with inflammatory stranding. These collections were new since compared to pre-treatment staging CT exams. While metastatic periaortic lymphadenopathy was considered in the differential diagnosis, the absence of a primary lesion or other metastases on PET/CT, supported an infective cause. Although patient A had a prior history of TB exposure, the lack of active TB elsewhere in the body, supported the diagnosis of MAA due to BCG therapy. Patient B had no history of prior TB exposure. Pathology from CT-guided biopsy of both cases was consistent with granulomatous infection secondary to their recent BCG therapy. M bovis from recent BCG therapy was cultured in patient A. In patient B, although the percutaneous biopsy did not yield M Bovis, it was cultured from the resected gross specimen.

Several different mechanisms have been postulated for the development of a MAA. These include infection of atherosclerotic intima, bacteremic spread through the vasa vasorum, contiguous spread from a gastrointestinal source, direct extension from vertebral osteomyelitis, or infection of a congenital abnormality such as aortic coarctation (Feigl & Feigl, 1986). In both our cases, there was no imaging evidence of tuberculosis elsewhere in the body. Blood cultures were negative for any systemic pathogens. Neither of the two patients were on chemotherapy or on any immunosuppressant drugs – both risk factors for the development of systemic tuberculous disease. Therefore, we suspect the thoracic and abdominal mycotic aneurysms in our respective patients were a result of M bovis contiguously spreading through the vasa vasorum resulting in thinning of the tunica intima, media and adventitia, and therefore resulting in mycotic aneurysms/pseudoaneurysms.

Surgical resection and debridement of infected aorta followed by an interposition graft or extra-anatomic bypass and long-term antibiotic therapy remain the gold standard management strategy (Muller et al., 2001). However, given the high post-surgical mortality ranging between 13.3–40% (Muller et al., 2001; Kyriakides et al., 2004), endovascular aortic repair (EVAR) remains a popular treatment of choice. Our first patient with an infected thoracic MAA was treated with a thoracic endovascular aortic repair (TEVAR), and the second one was treated with an open surgical resection along with an autologous venous bypass graft. Currently no consensus guidelines exist with respect to choosing EVAR versus open surgical repair. Therefore, a case by case decision needs to be made. Although EVAR has been shown to decrease the overall surgical morbidity and mortality (Kan et al., 2007), placing an endovascular graft in an infected vascular bed can lead to chronic graft infection. A recent multicenter trial demonstrated a 27% infection related complication in post-EVAR treatment of MAA despite continuous antibiotic treatment, with a 19% mortality rate from infection related complications (Sörelius et al., 2014). Given the multiple chronic medical conditions of our patient A, he was deemed a poor surgical candidate and was treated with TEVAR. He had an unremarkable post-procedural course and continues to be asymptomatic. His BCG treatment regimen is complete and has not received any BCG instillations since his TEVAR. Apart from his urothelial carcinoma, patient B was otherwise a healthy patient and a good surgical candidate. Open surgical repair with debridement of infected tissues and a reduction in infection in the vascular bed remains the gold standard for treatment. Patient B underwent surgical excision of the infected abdominal aorta with an autologous graft from the superficial femoral vein and continues to do well in the postoperative period with no relapse.

Given the relative rarity of MAA secondary to BCG treatment in the context of TCC, a complete understanding of BCGosis and best treatment options in these cases is limited. More work is required in understanding the pathophysiology of intravesical BCG causing aortitis as opposed to disseminated systemic BCGosis. Although no predisposing factors were identified in our patients that made them vulnerable to aortitis and MAAs, further investigation into genetic factors and predisposing aortic morphology, such as pre-existing atherosclerotic plaque is required. It is also important to note that the presentation of both these patients was remote from their last intravesical BCG instillation and speaks to the latent nature of BCG related infections. Post treatment, these index patients will be regularly followed up by urology, internal medicine, and vascular teams on a routine basis. The post-operative changes will be assessed with CT angiography and contrast enhanced ultrasound examinations on a routine basis.

## Conclusion

In summary, we report two patients that presented with MAAs secondary to M bovis from BCG treatment for TCC. The incidence of such vascular complications from BCG treatment is rare and very few cases have been reported in the literature. As both patients had varied medical profiles, different treatment options were pursued. These cases further expand the current knowledge on vascular complications related to BCG treatment. In the absence of formal guidelines, we recommend a multidisciplinary approach involving vascular surgery, diagnostic and interventional radiology, and infectious disease to manage these patients.
