# Correction to: Mycotic aortic aneurysms post-Intravesical BCG treatment for early-stage bladder carcinoma

**DOI:** 10.1186/s42155-019-0083-z

**Published:** 2019-12-16

**Authors:** Aman Wadhwani, Randy D. Moore, Darshan Bakshi, Anirudh Mirakhur

**Affiliations:** 1 Department of Radiology, University of Calgary, Calgary, AB Canada; 2 Division of Vascular Surgery, Department of Surgery, University of Calgary, Calgary, AB Canada; 3 Division of Interventional Radiology, Department of Radiology, University of Calgary, Calgary, AB Canada; 4 Foothills Medical Centre, G29, 1403-29th street NW, Calgary, AB T2N 2T9 Canada

**Correction to: CVIR Endovascular (2018) 1:28**

**https://doi.org/10.1186/s42155-018-0036-y**

In the published article (Wadhwani et al., 2018) the statement under the subheading ‘Consent for publication’ is incorrect.

“Case reports are exempt from the purview of our institutional health research ethics board and therefore no approval is required from the ethics board for publication.”

should read:

“Informed consent for publication of this case report and its accompanying images has been obtained from the patient”.
